# High isolation quad ports MIMO antenna loaded with FSS for 5G communication

**DOI:** 10.1038/s41598-025-05616-7

**Published:** 2025-06-20

**Authors:** Shaymaa M. Gaber, Radwa A. Kareem, Ahmed A. Ibrahim

**Affiliations:** 1 Faculty of Engineering, Egyptian Russian University, Badr, Egypt; 2 Communications and Electronics Engineering Department, October High Institute for Engineering and Technology, 6th of October City, Egypt; 3 Electronics and Communications Engineering Department, Minia University, El-Minia, Egypt; 4 Communications and Computer Engineering Department, Nahda University, Benisuef, Egypt

**Keywords:** MIMO antenna, Slot antenna, 5G applications, Wideband antenna, Frequency selective structure, Engineering, Electrical and electronic engineering

## Abstract

This paper introduces a high isolation quad ports MIMO (Multiple-Input Multiple-Output) antenna with a frequency selective surface (FSS) structure for higher frequency bands in 5G communication systems. The recommended antenna is designed for 28 GHz application. The single unit of the antenna consists of a microstrip feed line on one side and a rectangular slot in the ground plane on the other side. The four MIMO antennas are arranged orthogonally on a Rogers RO4003C substrate with a permittivity of 3.38, a loss tangent (tan δ) of 0.002, and a height of 0.203 mm. The substrate features a cross-shaped cut to enhance the isolation between ports. An FSS is placed beneath the MIMO system to improve the overall gain across the desired frequency band. The antenna size is 25.7 × 25.7 mm². The design and simulation of the proposed structure were carried out using CST MW Studio. The antenna with and without the FSS structure was constructed and tested to verify the simulation results. The results indicate that the suggested structure is worked from 25.5 GHz up to 30 GHz with insertion loss ≤ -22 dB and peak gain of around 8dBi. As well, the envelope correlation coefficient (ECC), diversity gain (DG), and channel capacity loss (CCL) are measured and achieved ≤ 0.002, ≥ 9.99 dB, ≤ 0.2 bit/s/Hz, respectively. Also, extra MIMO parameters such as mean effective gain (MEG), channel capacity, and total active reflection coefficient (TARC) are extracted and achieve good outcomes confirming the ability of the antenna to be applicable for 5G networks.

## Introduction

In today’s age of ubiquitous connectivity, wireless communication has become essential for the seamless operation of interconnected devices. From smartphones and laptops to IoT devices and industrial machinery, reliable wireless connectivity is critical. The performance of the wireless systems such as introducing high data rates, increasing connectivity, reducing latency, and increasing efficiency can be enhanced by utilizing the 5G technology^1^. The high band of the 5G introduces attenuation due to the atmospheric absorption which needs antennas with high gain to overcome this issue. Also, the transmission quality and the capacity of the system can be enhanced by using MIMO technology and increasing the antenna elements^2,3^. MIMO is an advanced antenna technology where both the transmitter and receiver are equipped with multiple antennas for wireless communication. Its popularity stems from its ability to minimize signal fading and ensure signal delivery, even in challenging environments such as urban or rural areas where signals may bounce off obstacles like trees and buildings before reaching their destination. Modern MIMO technologies are designed to provide ultra-low-power and cost-effective solutions^4–8^. Integrating multiple antenna units into a mobile phone with extremely limited space is a significant challenge.

Mutual coupling between antenna elements is a significant challenge in achieving high MIMO efficiency^9–13^. Minimizing this coupling is crucial to ensuring optimal performance and reliability in MIMO antenna systems. In^9^ Defected ground structures with E and rectangular shapes are used to decrease mutual coupling and improve MIMO performance for 4-port MIMO antenna designed for 5G applications with engineered ground defects. In^10^, low mutual coupling is achieved using a Defected Ground Structure (DGS) in combination with a two-element patch antenna featuring graphene radiators. In^11^, an investigation is conducted on high isolation using a 2-port fractal MIMO graphene antenna with rectangular partial ground planes to enhance isolation and return loss characteristics. To achieve isolation and good resonance match, ground stubs with an inverted L shape are deployed in the ground plane and the element is positioned orthogonally as discussed in^12^. In^13^, a graphene 2-port MIMO patch antenna with a RIS (Reconfigurable Intelligent Surface) structure for beam steering and a metamaterial cell to improve port isolation is suggested.

To satisfy the demanding specifications of 5G mobile communication systems, researchers have developed several high-gain antenna designs^14–19^. Gain enhancement can be achieved through a variety of methods, including array configuration^14,15^, An electromagnetic band gap (EBG)-based structure^16^, and an FSS-equipped four-element MIMO antenna has been suggested at 38.9 GHz for enhancement gain from 8dB to 10 dB, is presented in^17^. Broadband and high-gain characteristics are achieved using a circular patch featuring a circular slot and two via, combined with a partially reflective surface (PRS) loading as investigated in^18^.

This paper proposes a four-port MIMO antenna design for 5G-NR applications that uses frequencies between 25.5 and 30 GHz for operation and incorporates an FSS structure. Four-antenna MIMO system with orthogonally positioned elements and across-shaped cuts is examined for impedance and radiation characteristics. A Rogers RO4003C substrate, which is renowned for its high-frequency and low-loss performance is used to implement the antennas. An FSS is positioned beneath the MIMO system in order to improve overall gain throughout the target frequency band. CST microwave studio is used to design and simulate the suggested structure. The FSS-structured antenna design was manufactured and tested in order to confirm the outcomes of the simulation. The results indicate that the proposed structure is well-suited for 5G NR networks, as it aligns with the target frequency band. The new of this work is **first**, design of four four-port MIMO antenna with compact size and connected ground. **Second**, the antenna operation is performed from 25.5 GHz up to 30 GHz and with high isolation ≥ 22 dB. **Third**, the antenna is utilized FSS to increase the antenna’s gain up to 8 dBi.

## Single antenna

**Fig. 1:**
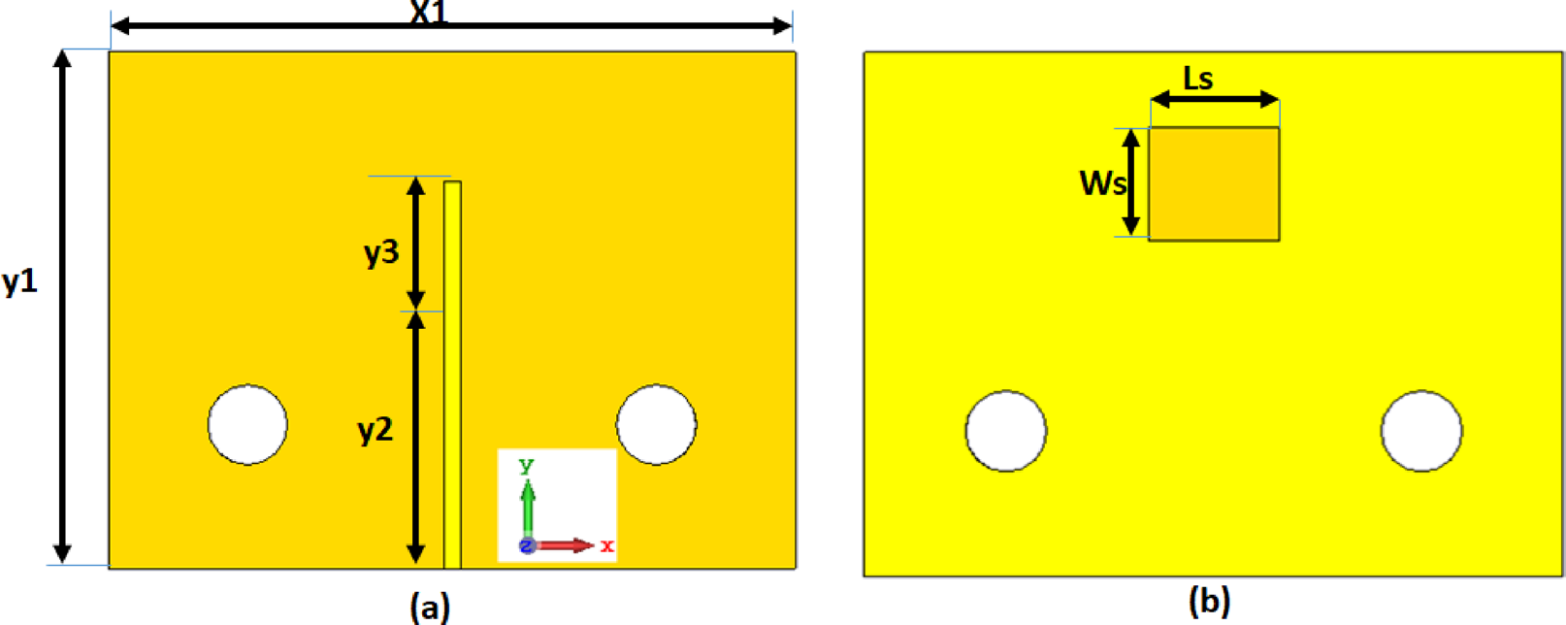
2D view of the slotted antenna with X1 = 16 mm, Y1 = 12 mm, y2 = 7 mm, y3 = 2 mm, Ls = 3 mm, Ws = 2.6 mm (**a**) Front view (**b**) Back view.

**Fig. 2:**
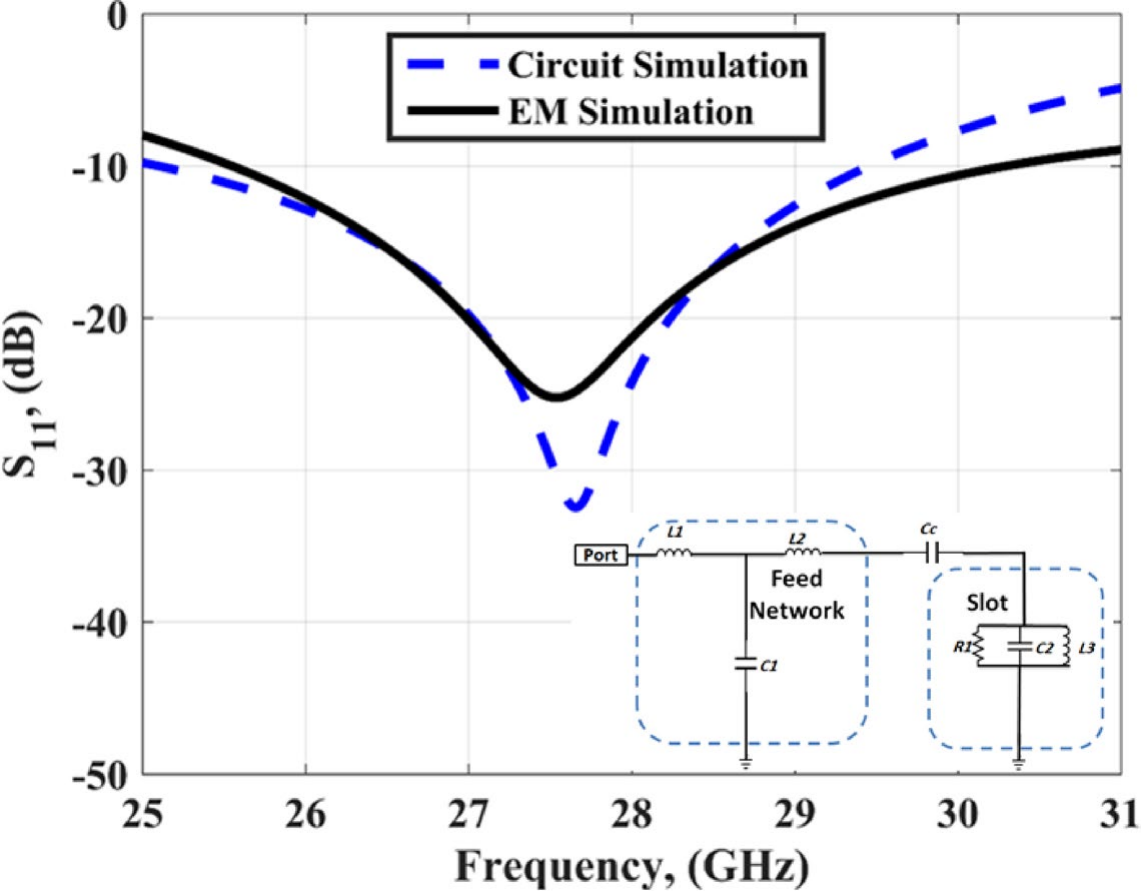
The S_11_ of the slotted antenna and *L1 = L2* = 0.259 nH, *C1* = 0.253pF, *Cc* = 0.745 pF, *R1* = 21.84 Ω, *C2* = 0.3684pF, *L3* = 0.6 nH.

The single antenna consists of a slot antenna with a microstrip line in front size and the slot is rectangular and etched from the back side as shown in Fig. 1. The microstrip line’s length is adjusted to achieve the operating frequency band. The antenna’s overall dimensions are 16 × 12 mm^2^ designed on a Rogers RO-4003 C substrate (εr = 3.38, tan δ = 0.0027), with a height of 0.203 mm. Figure 2 shows the simulated S_11_ outcomes extracted from the EM simulator and circuit simulation and the equivalent circuit model of the antenna. The slot antenna can be modeled as a parallel RLC circuit. The microstrip line can be modeled as two inductors and one capacitor as shown in Fig. 2. The antenna achieved a bandwidth changing from 25.5 to 30.5 GHz with S_11_≤-10 dB for the simulated outcomes while it achieved a bandwidth extended from 25 to 29.8 GHz with S_11_≤-10 dB for the circuit simulation outcomes with a good matching between the two results.

The slot length (Ls) equals λg/2 which is calculated using Eq. (1) from the resonant frequency (*f*_*r*_), speed of light (*c*) and the effective permittivity (є_reff_).

1 $$Ls=\frac{c}{{2{f_r}\sqrt {{\varepsilon _{reff}}} }}.$$

**Fig. 3:**
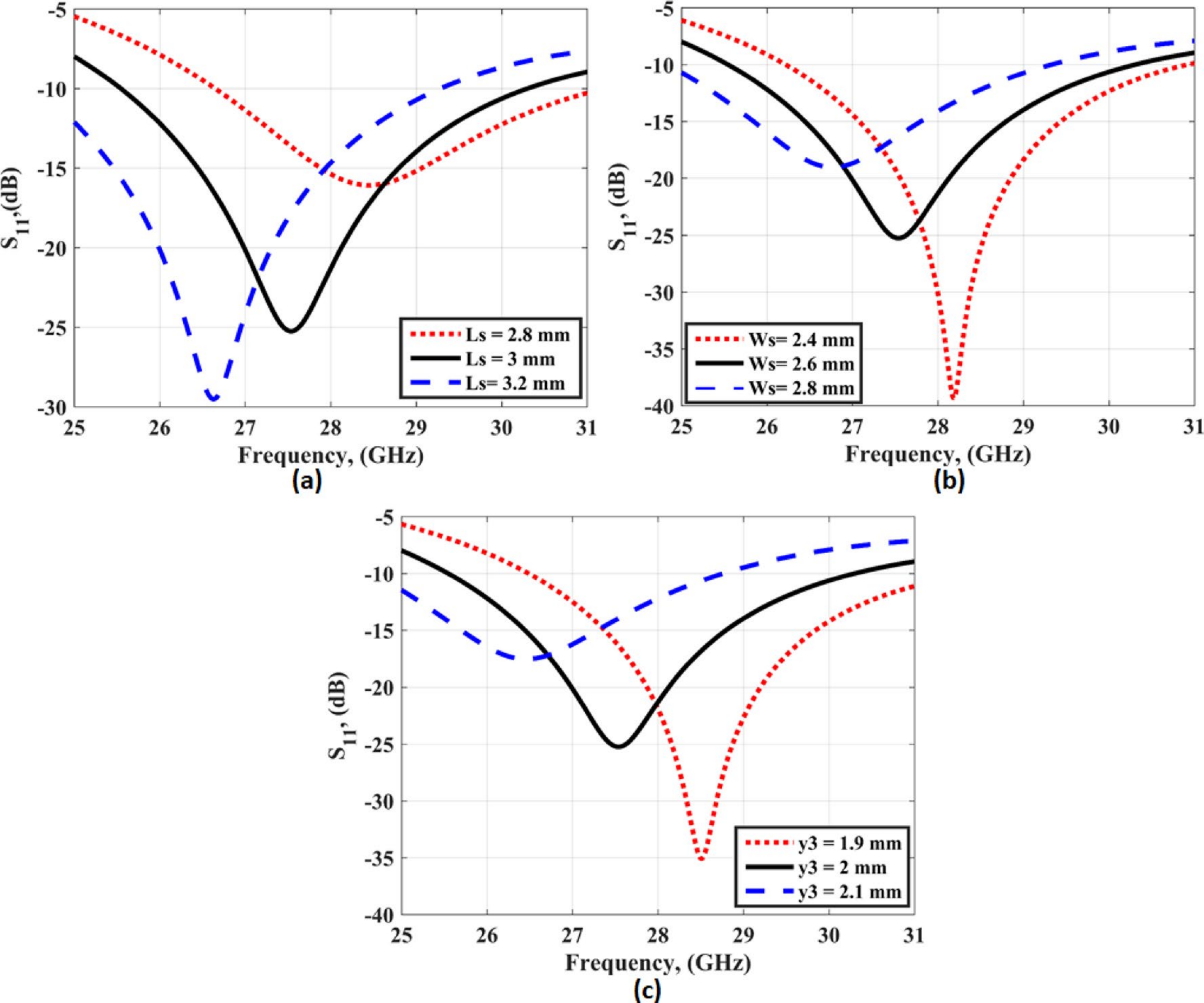
The S_11_ of the slotted antenna at different values of parameters (**a**) with changing of Ls (**b**) with changing of Ws (**c**) with changing of y3.

The Ws is optimized to achieve the best impedance matching at the resonant frequency. To study the effect of the main parameters (Ls, Ws, and y3) on the antenna reflection coefficient, parametric studies are investigated as illustrated in Fig. 3. As shown in Fig. 3(a), when the Ls equals 2.8 mm (Ws = 2.6 mm, y3 = 2 mm) the resonant frequency equals 28.8 GHz with S_11_≤-10 dB extended from 26.8 to 31 GHz. Also, when the Ls equals 3 mm the resonant frequency equals 27.5 GHz with S_11_≤-10 dB extended from 25.5 to 30.5 GHz. When the Ls equals 3.2 mm the resonant frequency equals 26.8 GHz with S_11_≤-10 dB extended from 25 to 29 GHz the Ls with a value of 3 mm is chosen for the antenna design. As illustrated in Fig. 3(b), when the Ws is increased from 2.4 to 2.8 GHz (Ls = 3 mm, y3 = 2 mm), the resonant frequency is slightly changed and the antenna impedance matched is affected. The Ws with a value of 2.6 mm is chosen for the antenna design. As shown in Fig. 3(c), when the y3 equals 1.9 mm (Ls = 3 mm, Ws = 2.6 mm) the resonant frequency equals 28.5 GHz with S_11_≤-10 dB extended from 26.5 to 31 GHz. Also, when the y3 equals 2 mm the resonant frequency equals 27.5 GHz with S_11_≤-10 dB extended from 25.5 to 30.5 GHz. When the y3 equals 2.1 mm the resonant frequency equals 26.3 GHz with S_11_≤-10 dB extended from 25 to 28.8 GHz the y3 with a value of 2 mm is chosen in antenna design.

**Fig. 4:**
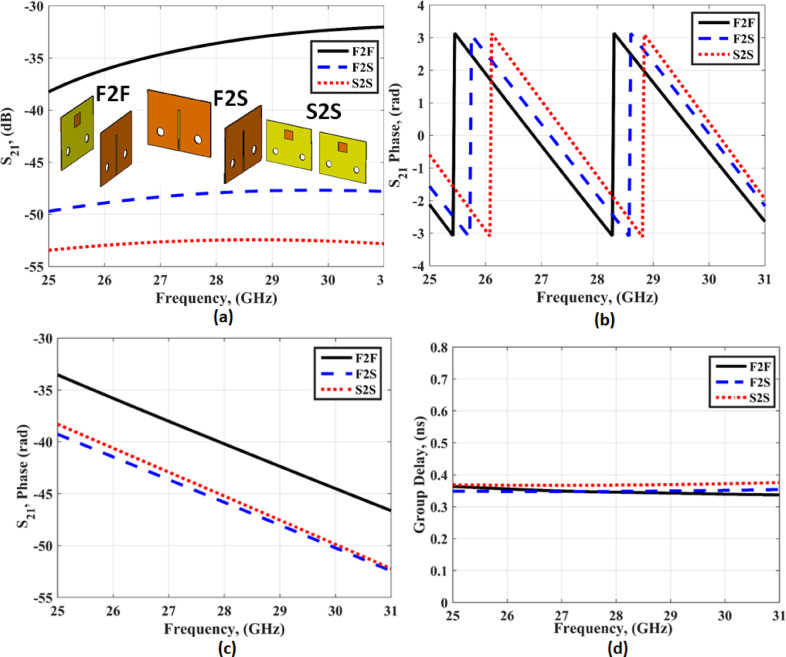
The three configurations outcomes (**a**) S_21_ (**c**) S_21_ phase (**b**) S_21_ unwrapped phase (**d**) Group delay.

The time domain investigation is utilized to this antenna to confirm its good performance. Four parameters as shown in Fig. 4 are studied to show the ability of the antenna to operate in a good way in the high frequency and wideband operation. Three configurations are studied as illustrated in Fig. 4(a). The two antennas are simulated with a distance of 70 mm between them. The four parameters are extracted for the three configurations as shown in Fig. 4. It is seen that, the S_21_ ≤ 30dB is achieved four the three configuration as shown in Fig. 4(a). As well, the S_21_ phase has linear feature in the three configuration at the desired frequency band as shown in Fig. 4(b), (c). Finally, the group delay at 0.36 ns with very small variation around this value is achieved for the three configuration as shown in Fig. 4(d).

Also, the quality of the received signal can be evaluated by the system fidelity factor (SFF)^19^. The SFF can be extracted from Eq. (2)

2 $$SFF=\mathop {\hbox{max} }\limits_{n} \left( {\frac{{\int\limits_{{ - \infty }}^{\infty } {{T_s}\left( t \right){R_s}\left( {t+\tau } \right)d\tau } }}{{\begin{array}{*{20}{c}} {\left( {\sqrt {{{\int\limits_{{ - \infty }}^{\infty } {\left| {{T_s}\left( t \right)} \right|} }^2}dt} } \right)}&{\left( {\sqrt {{{\int\limits_{{ - \infty }}^{\infty } {\left| {{R_s}\left( t \right)} \right|} }^2}dt} } \right)} \end{array}}}} \right).$$

where Ts(t) is the transmitted excitation signal, Rs(t) is the radiated signal in the received antenna, H(ω) is the system transform function, and τ denotes the shifted variable. The simulated SSF equals 0.982, 0.954, and 0.842 in the F2F, F2S, and S2S, respectively. From the time domain analysis, it is clear that the antenna can receive pulses without distortion to enable it to be operated in high frequency and wide band communication.

## The 4 elements MIMO antenna

**Fig. 5:**
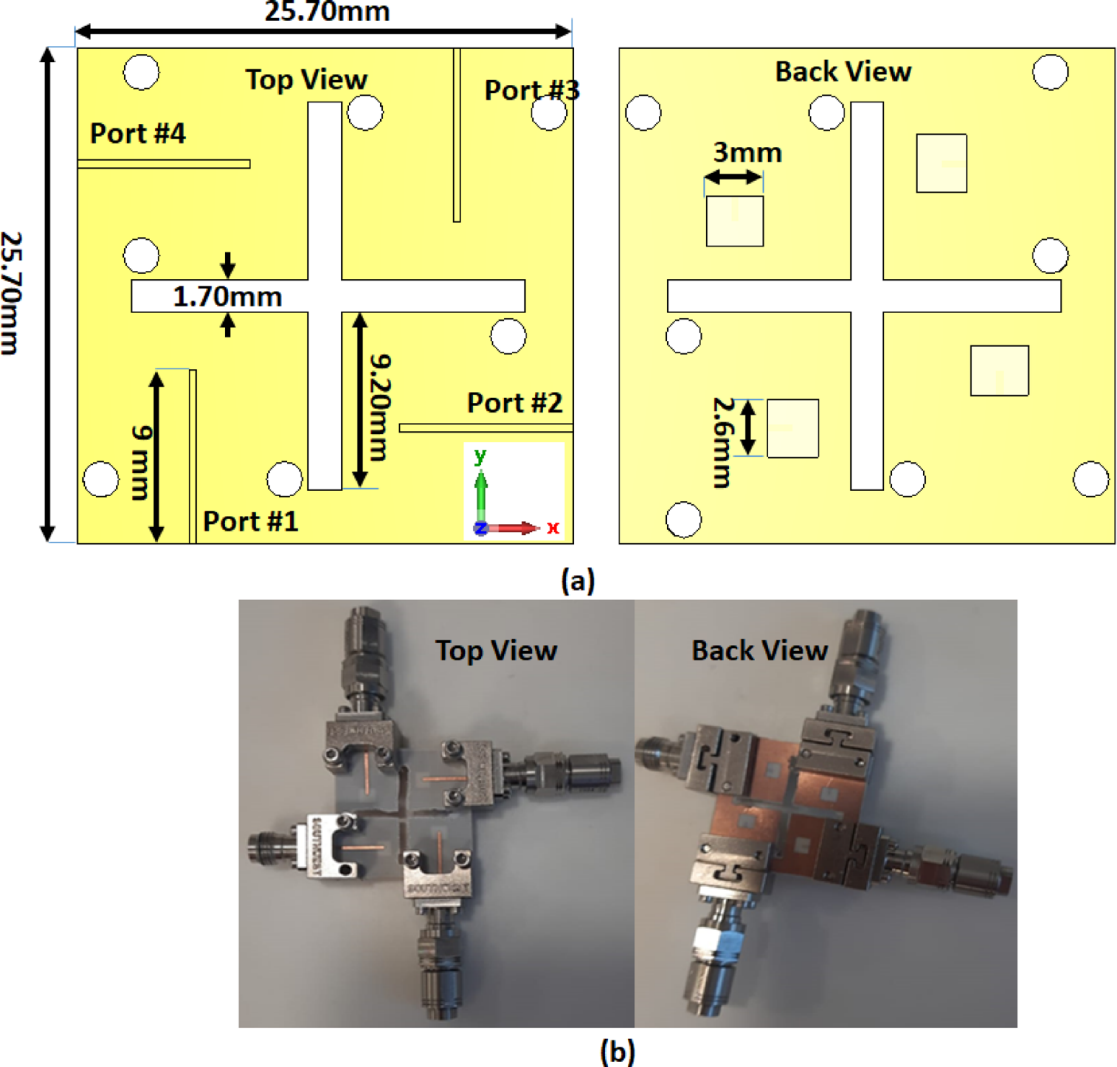
Configuration of the slotted MIMO antenna, (**a**) 2D view (**b**) antenna prototype.

**Fig. 6:**
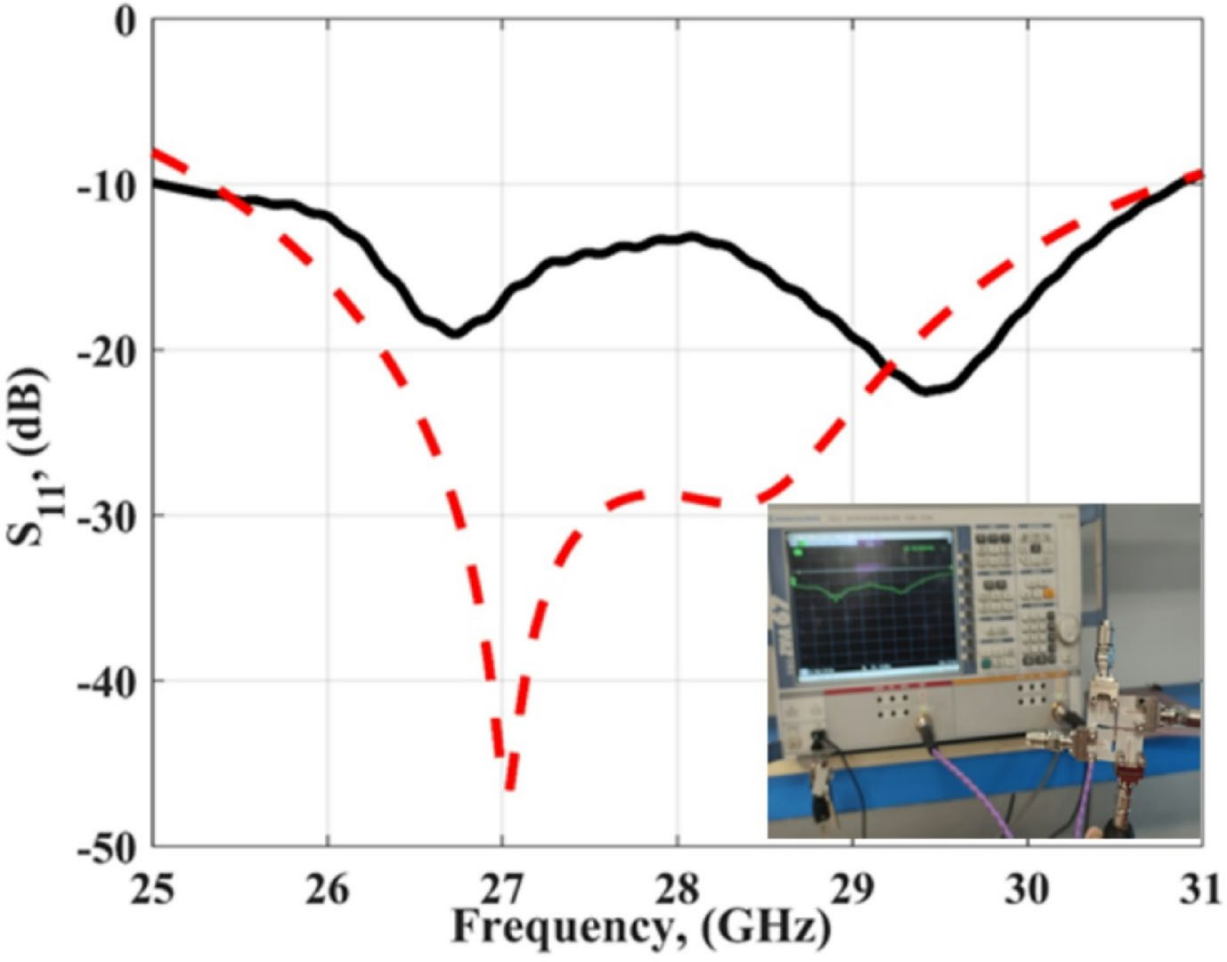
The S_11_ of the slotted MIMO antenna.

The configuration of the proposed MIMO antenna structure is shown in Fig. 5. The antenna’s overall dimensions are 25.7 × 25.7 mm^2^ designed on a Rogers RO-4003 C substrate (εr = 3.38, tan δ = 0.0027), with a height of 0.203 mm. The single unit is repeated four times to achieve the MIMO antenna structure. The four elements are organized to be perpendicular to each other and a cross-shaped cut from the substrate is made to enhance the isolation between the elements without increasing antenna size as illustrated in Fig. 5(a). The slotted MIMO antenna has been manufactured, and the prototype model is also displayed in Fig. 5 (b). Figure 6 shows the ZVA-67 (VNA) for testing the antenna.

**Fig. 7:**
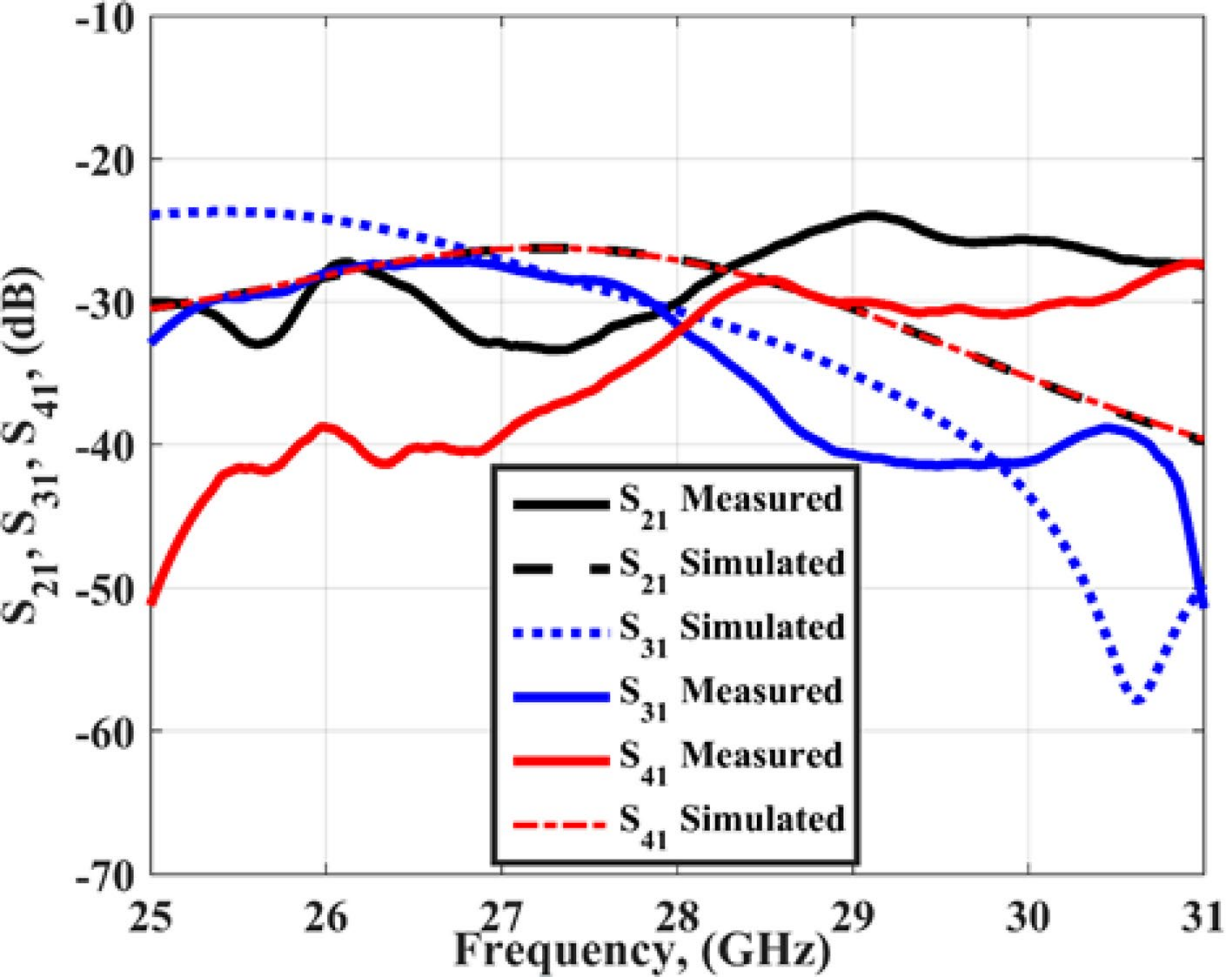
The insertion losses of the slotted MIMO antenna.

Figure 6 shows the measured and simulated S_11_ outcomes at port 1. The antenna achieved a bandwidth changing from 25.5 to 31 GHz with S_11_≤-10 dB for the simulated outcomes while it achieved a bandwidth extended from 25 to 31 GHz with S_11_≤-10 dB for the tested outcomes. The VNA screenshot is added in Fig. 6. The insertion losses (S_21_, S_31_, S_41_) of the MIMO antenna are introduced in Fig. 7. As shown in the figure, all the measured and simulated transmission coefficients are below − 22 dB, which indicates that the good isolation between the elements and accepted.

**Fig. 8:**
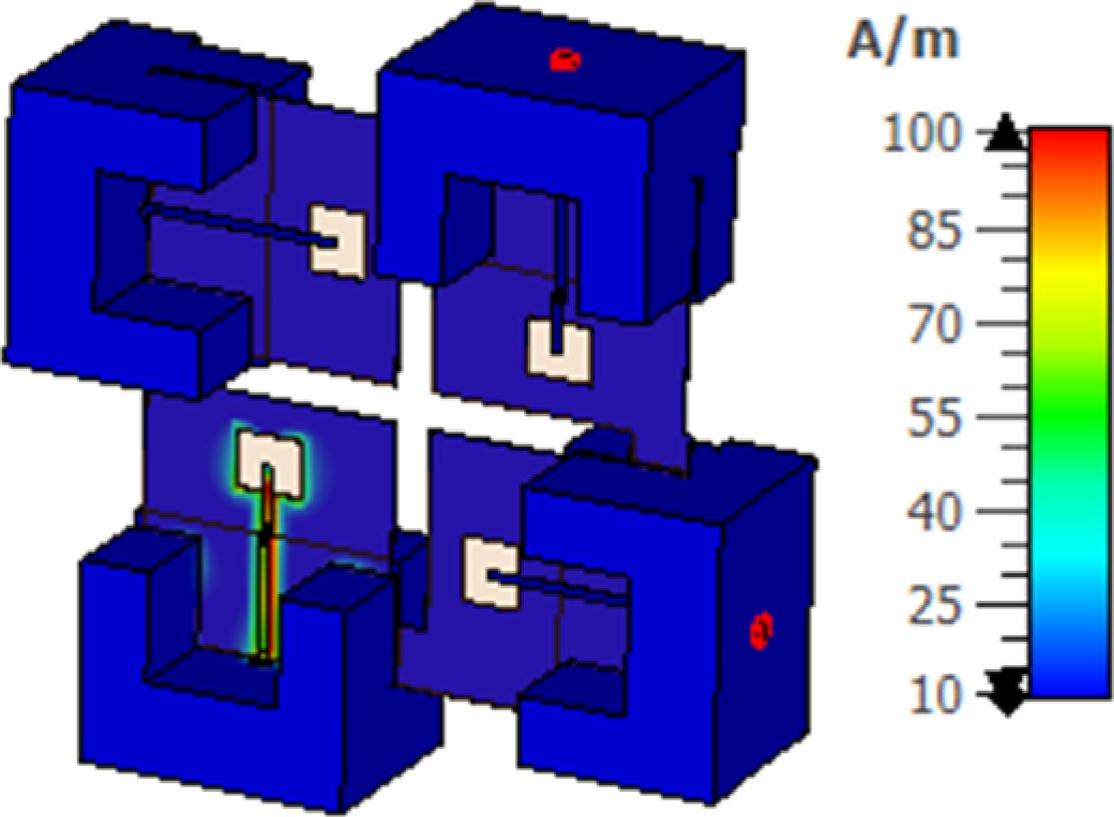
The current distribution of the structure at pot 1.

**Fig. 9:**
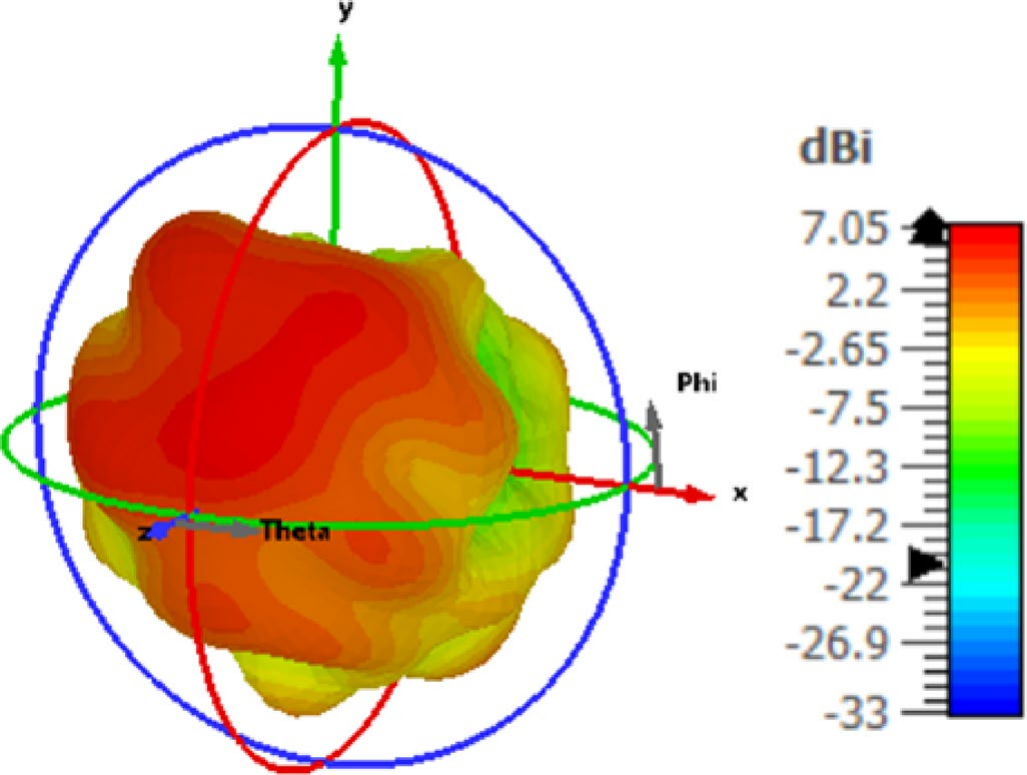
The 3D gain of the structure at pot 1.

The antenna’s current distribution at 28 GHz and port 1 is displayed in Fig. 8. As appears in the figure, the majority of current is collected around the slot of the antenna 1 and there is a very little current transferred to other ports which confirms the very good isolation between all ports. The 3D gain of the MIMO structure at port 1and at 28 GHz is illustrated in Fig. 9. The gain equals 7.05 dBi at 28 GHz. The MIMO antenna is added inside the anechoic chamber for measuring the radiation pattern. The horn antenna which is considered a reference antenna operated at a bandwidth extended from 26 GHz up to 40 GHz is worked as a transmitter while the MIMO antenna is placed at the receiving end with 70 cm distance from the horn antenna to ensure the far filed region criteria. The MIMO antenna is rotated in both x-z and y-z planes to measure the radiation pattern features. The MIMO antenna is attached to the VNA’s port 1, and the horn antenna is attached to the other port to measure the S21 to calculate the gain of the antenna from it^20^.

**Fig. 10:**
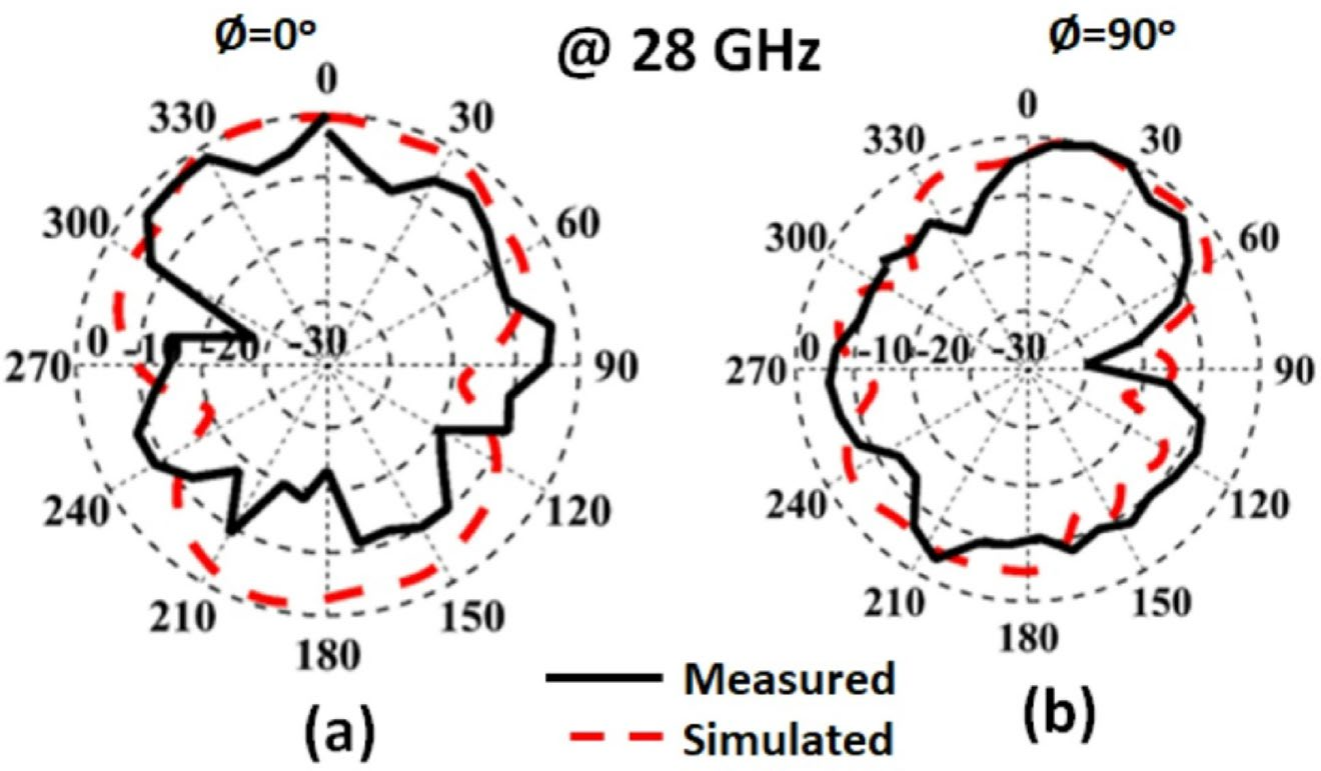
The normalized pattern.

**Fig. 11:**
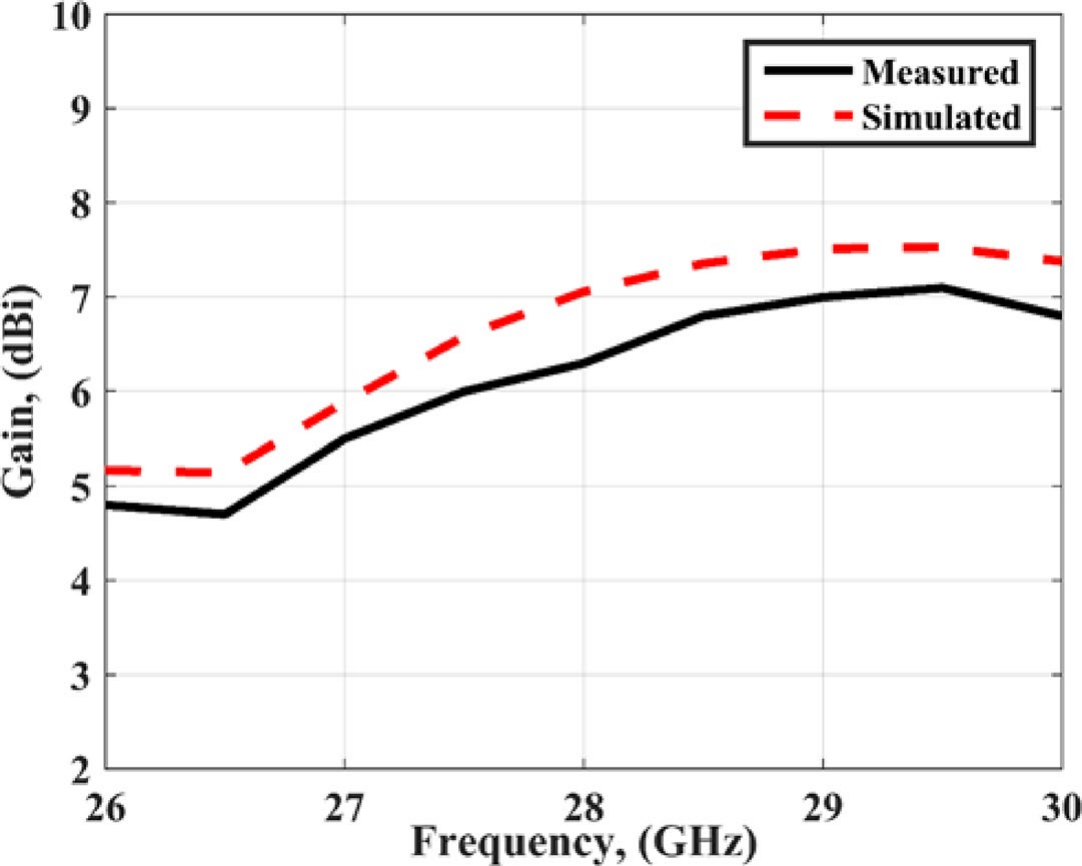
The peak gain.

The measured and simulated radiation characteristics of the suggested MIMO structure in the x-z and y-z planes at 28 GHz are shown in Fig. 10. The antenna achieved bidirectional patterns due to the slot in the ground plane with a good matching between the simulated and measured. It is seen that there is a small shift between the two outcomes which is due to the large connector size which affected the radiation characteristic. Figure 11 shows the peak gain of the slotted MIMO antenna versus frequency. It is noticed that the MIMO structure has a peak gain of 7.05 dBi and 6.2dBi from the simulated and measured results respectively 28 GHz.

## The FSS unit cell

**Fig. 12:**
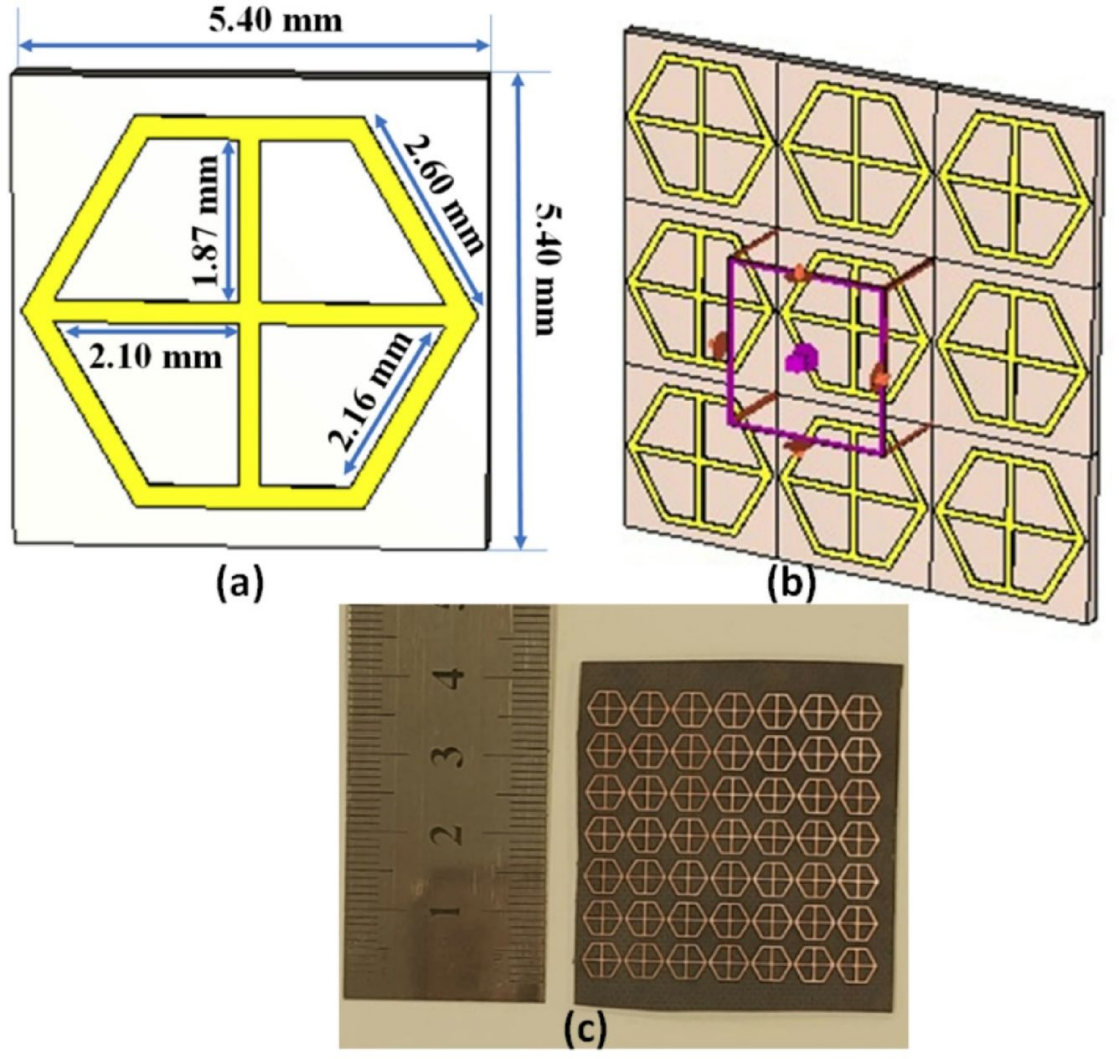
The FSS structure (**a**) 2D view (**b**) Boundary condition (**c**)7 × 7 prototype.

Figure 12 (a) displays the proposed unit cell by using FSS to increase the antenna gain. The FSS consists of a conducting hexagonal-shaped bounded cross-shaped conductor on the front view and there is no conducting material on the other side with an overall size of 5.4 × 5.4 mm^2^as illustrated in Fig. 12.This structure is printed on a substrate (Rogers RT-5880) with a relative permittivity of ε_r_ = 2.2 and a thickness of 0.508 mm.

**Fig. 13:**
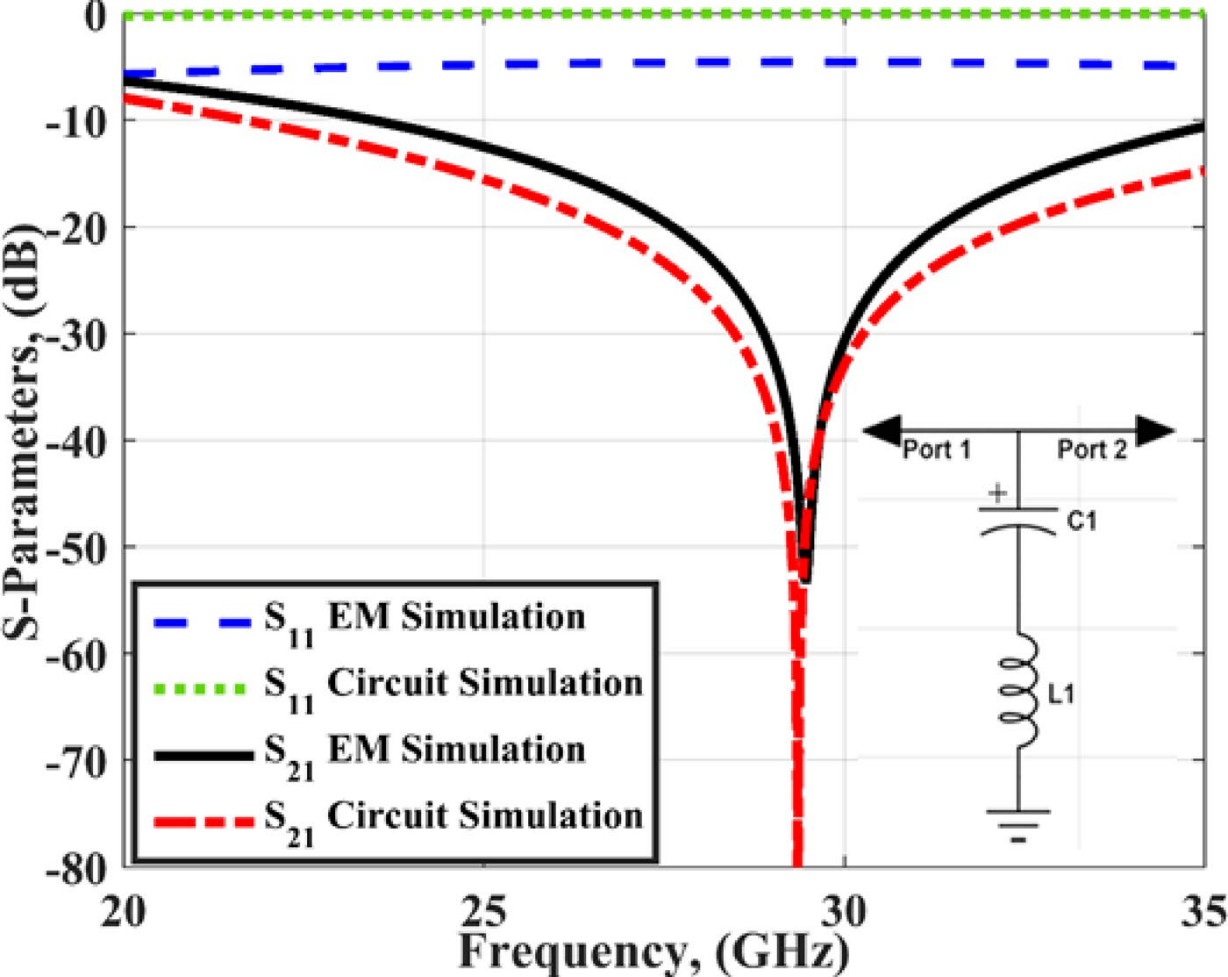
The return loss of the FSS unit cell.

The reflection–based FSS which is placed below the antenna is utilized in this design to reflect the radiation from the back and increase the antenna gain. The FSS unit cell is simulated using the CST software by assigning a unit cell boundary condition as illustrated in Fig. 12(b). As well the Floquet ports is utilized to excite the unit cell in the ± z directions. The prototype of the FSS with 7 × 7 cells is shown in Fig. 12(c). The FSS unit cell is simulated and the S-parameters are extracted and displayed in Fig. 13. The unit cell behaves as a band stop filter with insertion loss ≤-10 dB from 24 GHz up to 35 GHz. This means, that when the FSS cells are added below the antenna the wave is reflected from the cells and behaves as a reflector, and then the antenna gain will be improved. As well, the FSS unit cell can be modeled as an inductor in series with a capacitor as shown in Fig. 13 with inductance (L = 0.2674 nH) and capacitance of (C = 0.1099 pF). The S-parameters extracted from the EM simulator and circuit simulator are compared and achieved a good level of matching as illustrated in Fig. 13.

## Complete antenna structure

**Fig. 14:**
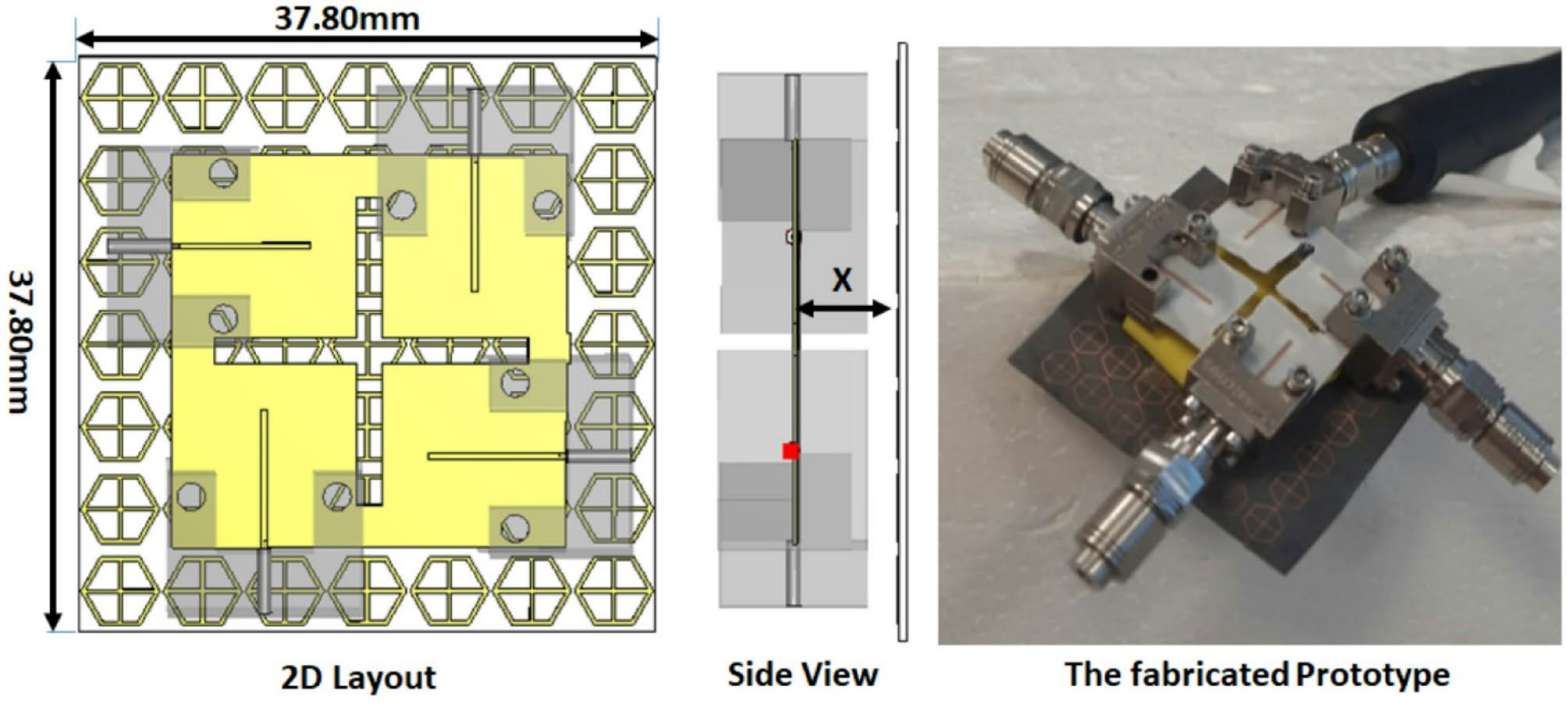
Set up of the slotted MIMO antenna integrated with the FSS.

**Fig. 15:**
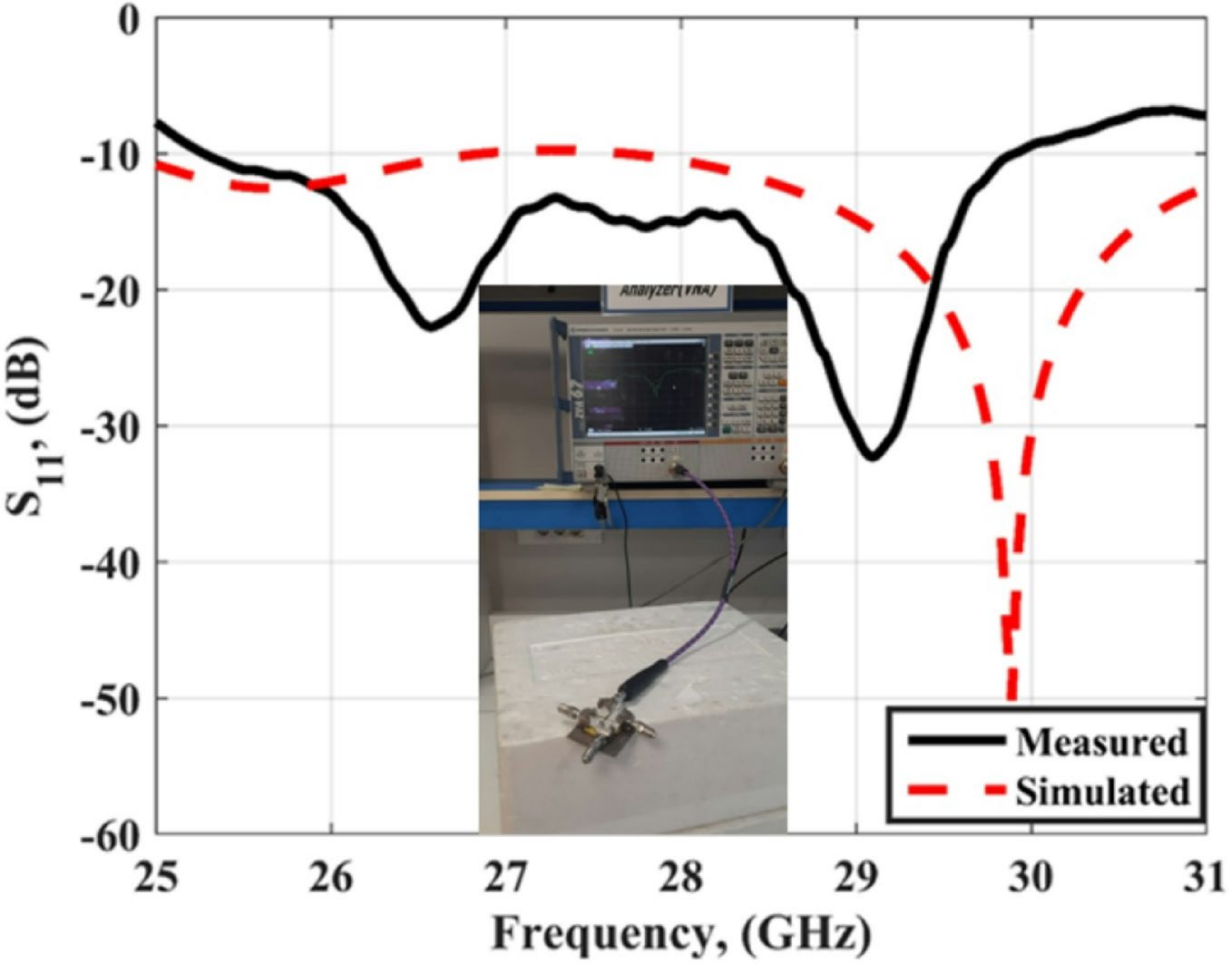
S_11_ of the MIMO structure integrated with the FSS.

**Fig. 16:**
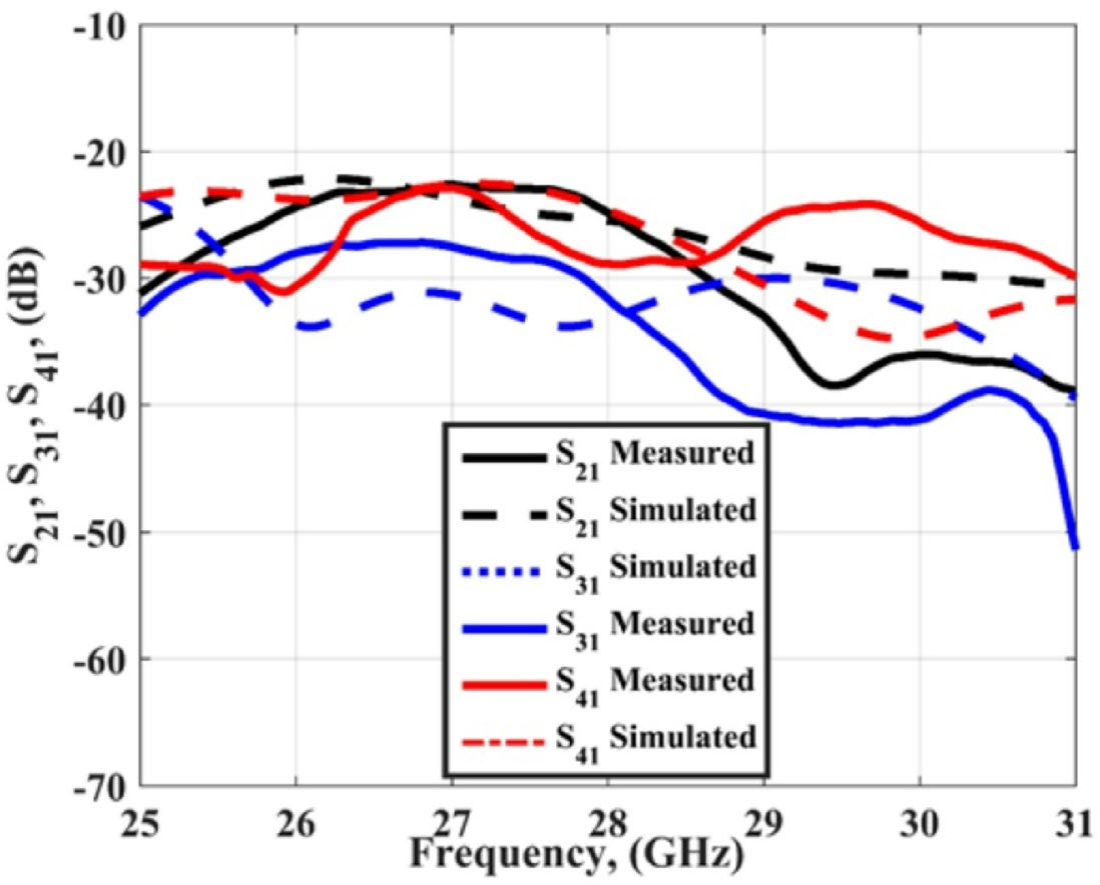
The insertion losses of the MIMO structure loaded with the FSS.

The slotted MIMO antenna discussed in the previous section is loaded with FSS cells to produce the proposed antenna. The FSS cells are positioned beneath the antenna to behave as reflectors and enhance the antenna gain as shown in Fig. 14. The FSS are placed at a distance (X) which is optimized to attain the intended matching and antenna gain. The optimized distance of X = 5.7 mm which is the minimum chosen distance because of the connector size. The setup of the proposed antenna and its fabricated prototype is shown in Fig. 14. A 7 × 7 FSS cells with overall size of 37.8 mm×37.8 mm are chosen to produce the desired features. The FSS cell size has positive effect on the antenna gain, larger cell size produce larger antenna gain but it increased the antenna size. So, a balance between antenna size and gain should be considered. A 5.7 mm polystyrene (foam layer) with 1.03 dielectric constant is utilized to differentiate between the MIMO antenna and the FSS array. The antenna is tested using a Vector Network Analyzer (VNA) (ZVA-67) as shown in Fig. 15. Figure 15 shows the measured and simulated S_11_ outcomes at port 1. The antenna achieved a bandwidth changing from 25 to 31 GHz with − 10dB for the simulated outcomes while it achieved a bandwidth changing from 25.5 to 30 GHz with − 10dB for the tested outcomes. The VNA screenshot is added in Fig. 15. The insertion losses (S_21_, S_31_, S_41_) of the MIMO antenna are introduced in Fig. 16. As shown in the figure, all the measured and simulated transmission coefficients are below − 22 dB, which indicates that the isolation between the elements is very good and accepted. The simulated results are matched in a good level with small shift between them. This is because of the fabrication tolerance, and the misalignment of the FSS cells.

**Fig. 17:**
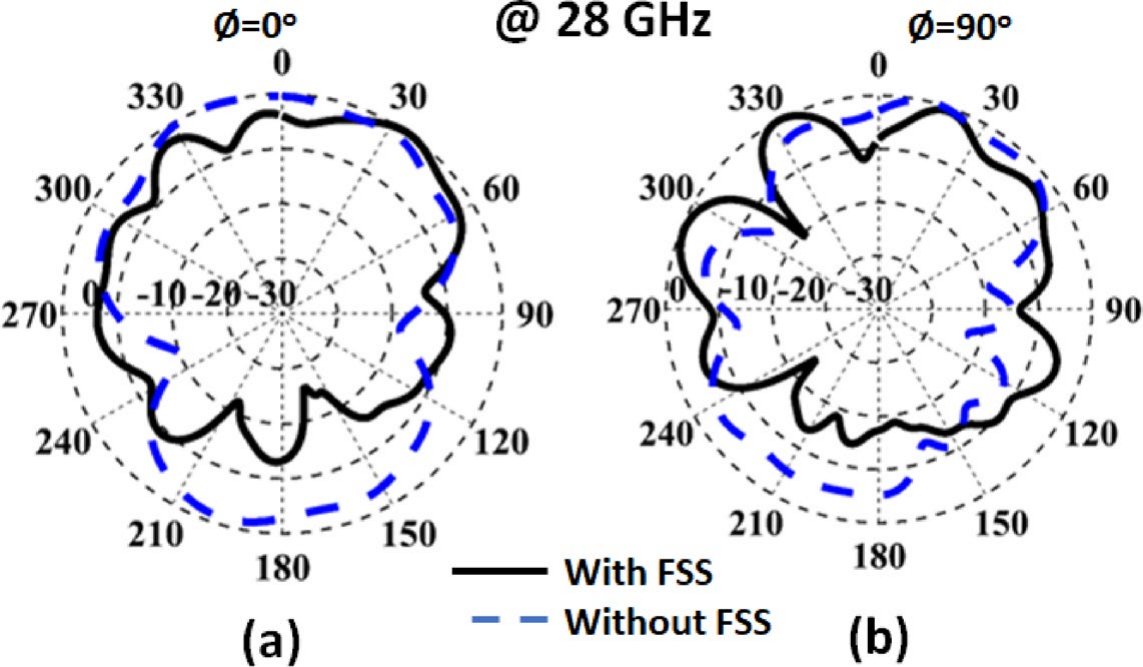
The normalized pattern with/without FSS.

Figure 17 illustrates the pattern in the two planes of the antenna with and without FSS cells. It is seen that the FSS enhances the antenna’s radiation characteristic and decreases its back radiation which in turn enhances the antenna gain.

**Fig. 18:**
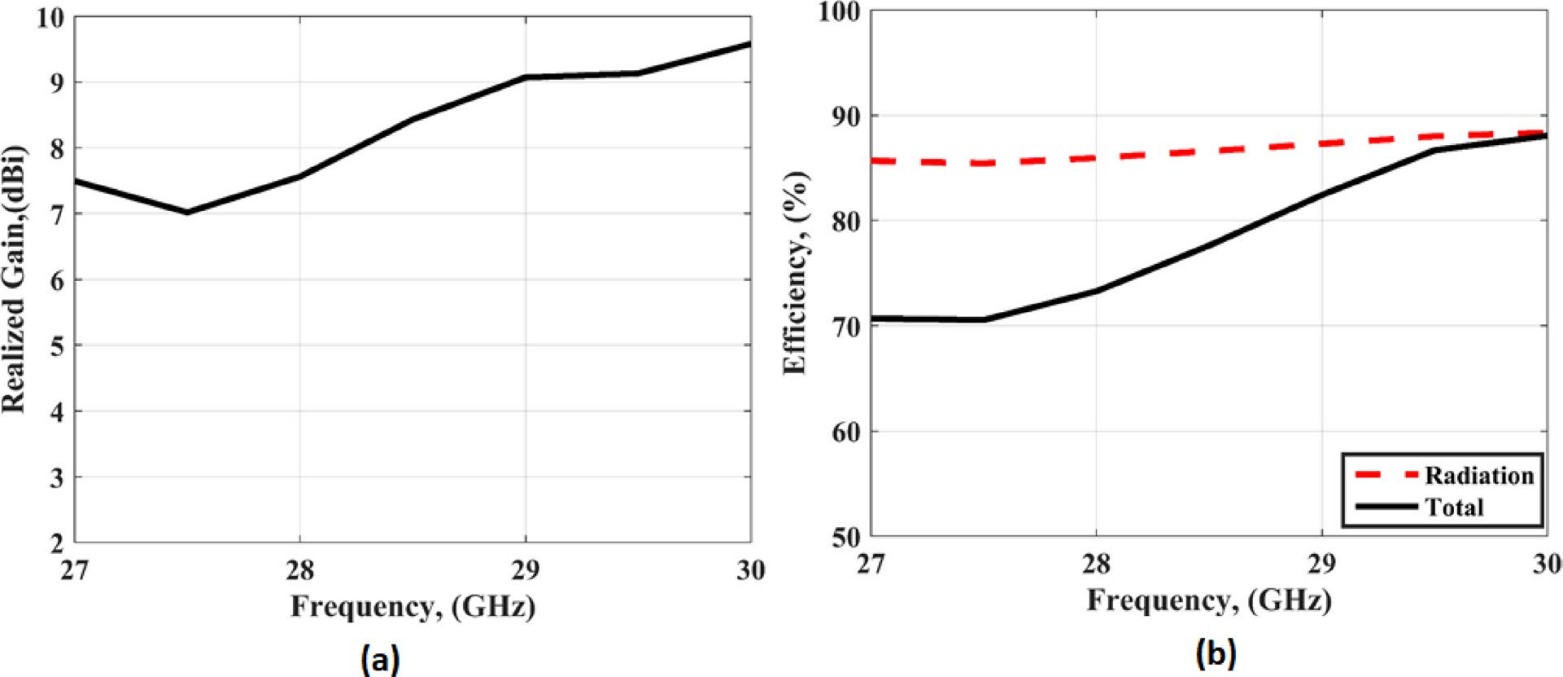
(**a**) The realized gain (**b**) The efficiency.

Figure 18 (a) shows the realized gain of the recommended antenna. It is seen that the antenna has simulated a realized gain of 7.8 dBi at 28 GHz. Figure 18(b) illustrates the antenna radiation and total efficiency. The antenna has a radiation efficiency of around 88% and a total efficiency ranging from 70 to 88%. The reduction of the total efficiency is due to the increasing the conducting and dielectric materials in the FFS layer.

**Fig. 19:**
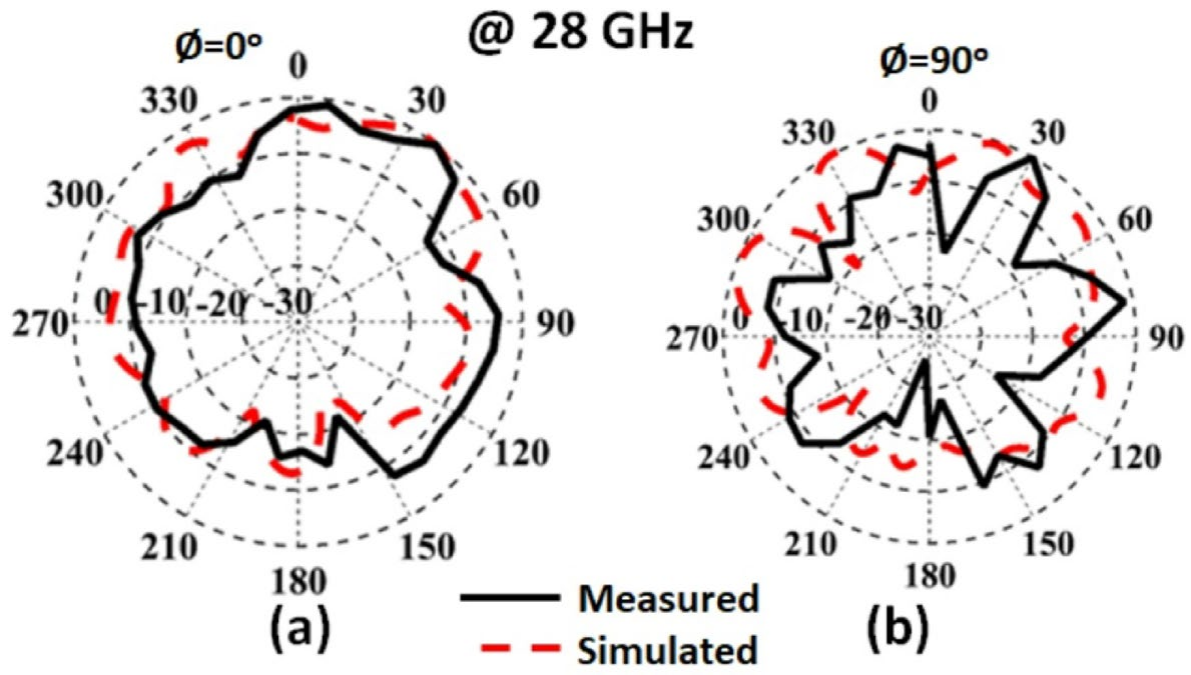
The normalized pattern.

**Fig. 20:**
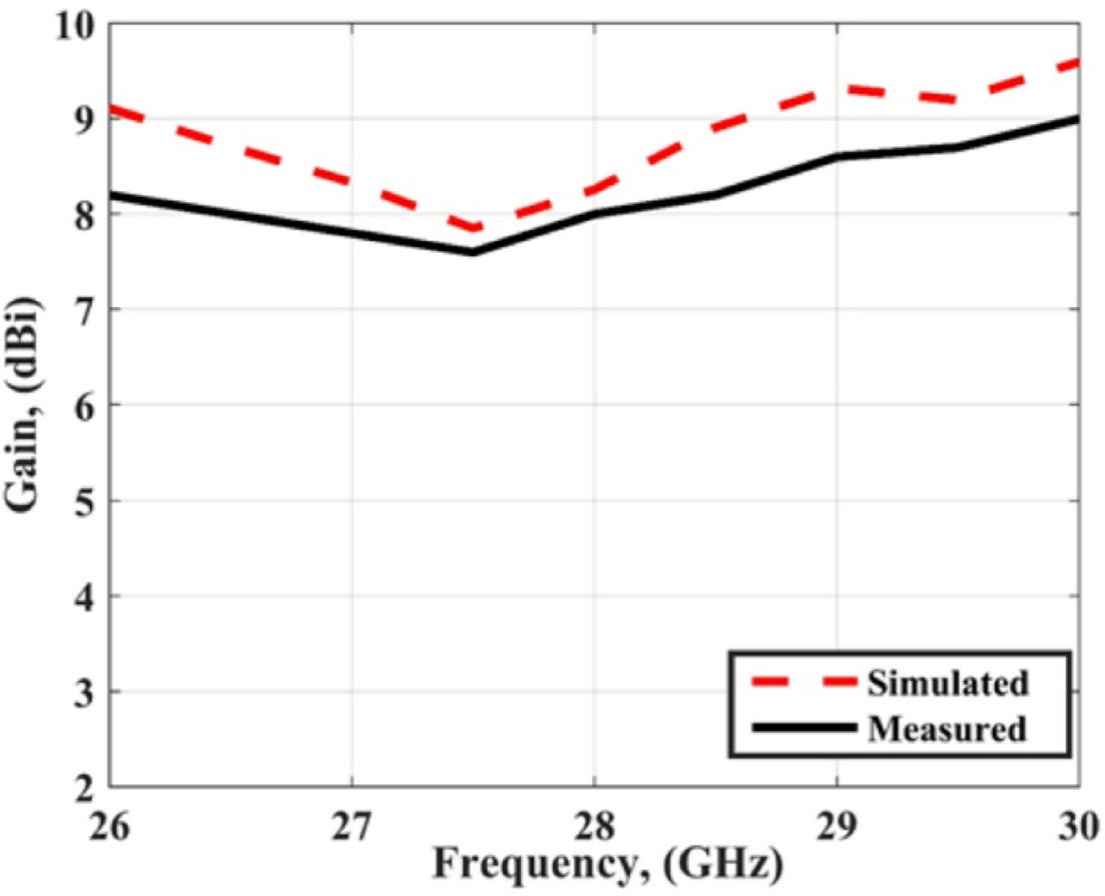
The peak gain.

The radiation patterns are extracted using the same steps in the previous section. The measured and simulated radiation characteristic of the suggested MIMO structure with FSS in the x-z and y-z planes at 28 GHz are shown in Fig. 19. The antenna achieved semi-unidirectional patterns due to the FSS cells with a good matching between the simulated and measured results. It is seen that there is a small shift between the two outcomes which is due to the large connector size which affected the radiation pattern. Figure 20 shows the peak gain of the slotted MIMO antenna with FSS versus frequency. It is noticed that the MIMO structure has a max gain of 8.05 dBi and 8dBi from the simulated and tested results at 28 GHz respectively. The conclusion can be drawn from the FSS cells can enhance the antenna gain with around 2dBi more than the antenna without FSS.

## MIMO parameters

The ECC, DG, CCL, TARC, MEG and Channel capacity are the main indicators to determine the MIMO performance. The ECC with small value is good thing because it reflects the correlation between cells. The ECC can be extracted from radiation characteristic or return loss as Eqs. (3) and (4)^21,22^.

3 $$ECC={\rho _e}=\frac{{{{\left| {\int {\int {_{{4\Pi }}\left[ {{F_1}\left( {\theta ,\varphi } \right) \bullet {F_2}\left( {\theta ,\varphi } \right)d\Omega } \right]} } } \right|}^{\text{2}}}}}{{\int {\int {_{{4\Pi }}{{\left| {{F_1}\left( {\theta ,\varphi } \right)} \right|}^2}d\Omega \int {\int {_{{4\Pi }}{{\left| {{F_2}\left( {\theta ,\varphi } \right)} \right|}^2}d\Omega } } } } }}$$

4 $$ECC={\rho _e}(i,j,N)=\frac{{{{\left| {\sum\limits_{{n=1}}^{N} {S_{{i,n}}^{*}{S_{n,j}}} } \right|}^2}}}{{{\prod _{k=i,j}}\left[ {1 - \sum\limits_{{n=1}}^{N} {S_{{k,n}}^{*}{S_{n,k}}} } \right]}}.$$

**Fig. 21:**
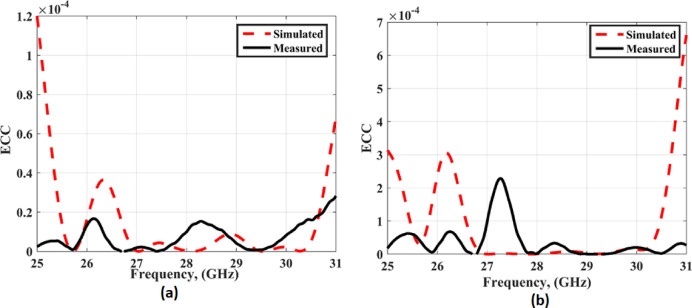
The ECC outcomes (**a**) without FSS (**b**)with FSS.

Figure 21 shows the value of the ECC is below 0.0002 in both cases with and without FSS through the entire bandwidth which confirms the high isolation and low correlation between antenna elements.

The relation between the DG and ECC is introduced in Eq. (5)^21^

5 $$\:\text{D}\text{G}=10\times\:\sqrt{1-{\left|\text{E}\text{C}\text{C}\right|}^{2}}.$$

**Fig. 22:**
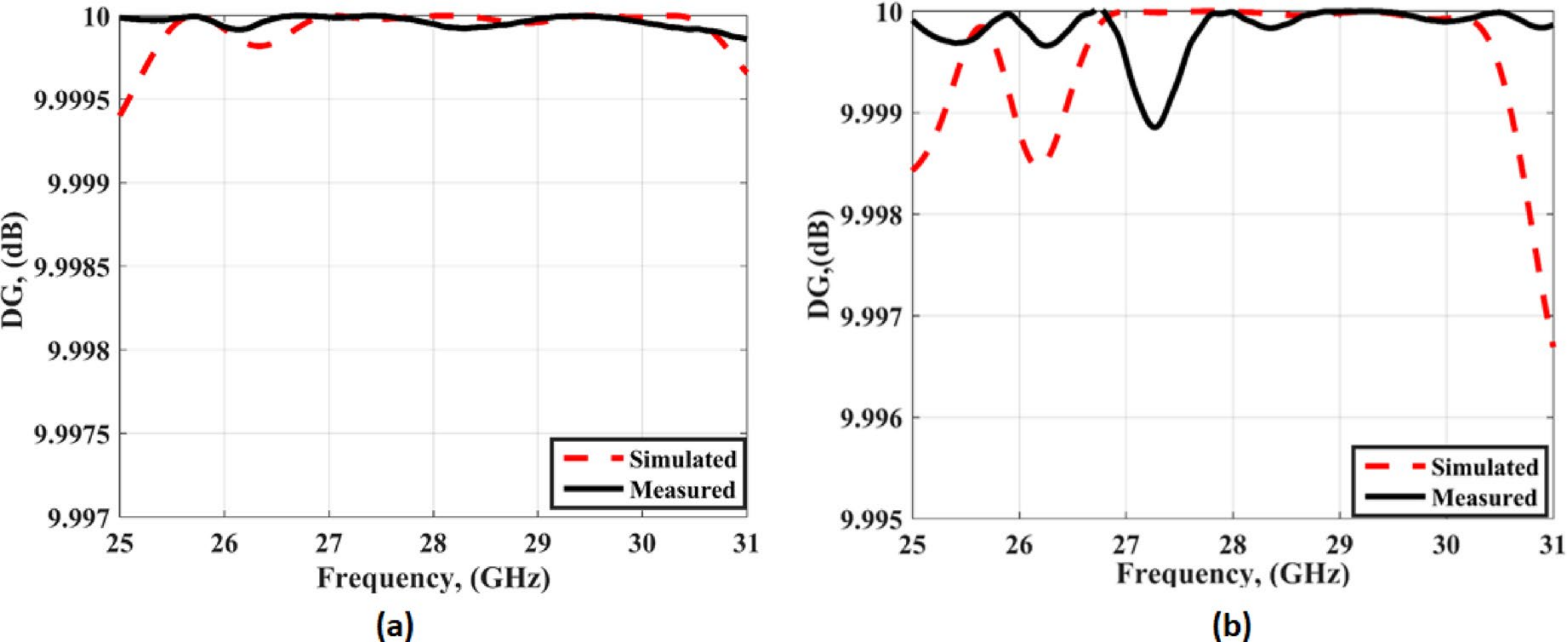
The DG outcomes (**a**) without FSS (**b**)with FSS.

The DG is achieved a result higher than 9.99 dB in both cases with and without FSS within he operating frequency range as displayed in Fig. 22.

The channel capacity loss (bit/s/Hz) is the third indicator and can be extracted using Eqs. (6), (7)^22,23^.

6 $$C(Loss)= - {\log _2}\det ({\psi ^R})$$

7 $$\begin{gathered} {\psi ^R}=\left[ {\begin{array}{*{20}{c}} {{\rho _{11}}}&{{\rho _{12}}}&{{\rho _{13}}}&{{\rho _{14}}} \\ {{\rho _{21}}}&{{\rho _{22}}}&{{\rho _{23}}}&{{\rho _{24}}} \\ {{\rho _{31}}}&{{\rho _{32}}}&{{\rho _{33}}}&{{\rho _{34}}} \\ {{\rho _{41}}}&{{\rho _{42}}}&{{\rho _{43}}}&{{\rho _{44}}} \end{array}} \right],{\rho _{ii}}=1 - \sum\limits_{{n=1}}^{4} {{{\left| {{S_{in}}} \right|}^2}} \hfill \\ and \hfill \\ {\rho _{ij}}= - \left| {\sum\limits_{{n=1}}^{4} {S_{{in}}^{*}{S_{nj}}} } \right|,for\begin{array}{*{20}{c}} {}&{} \end{array}i,j=1,2,3or4 \hfill \\ \end{gathered} .$$

**Fig. 23:**
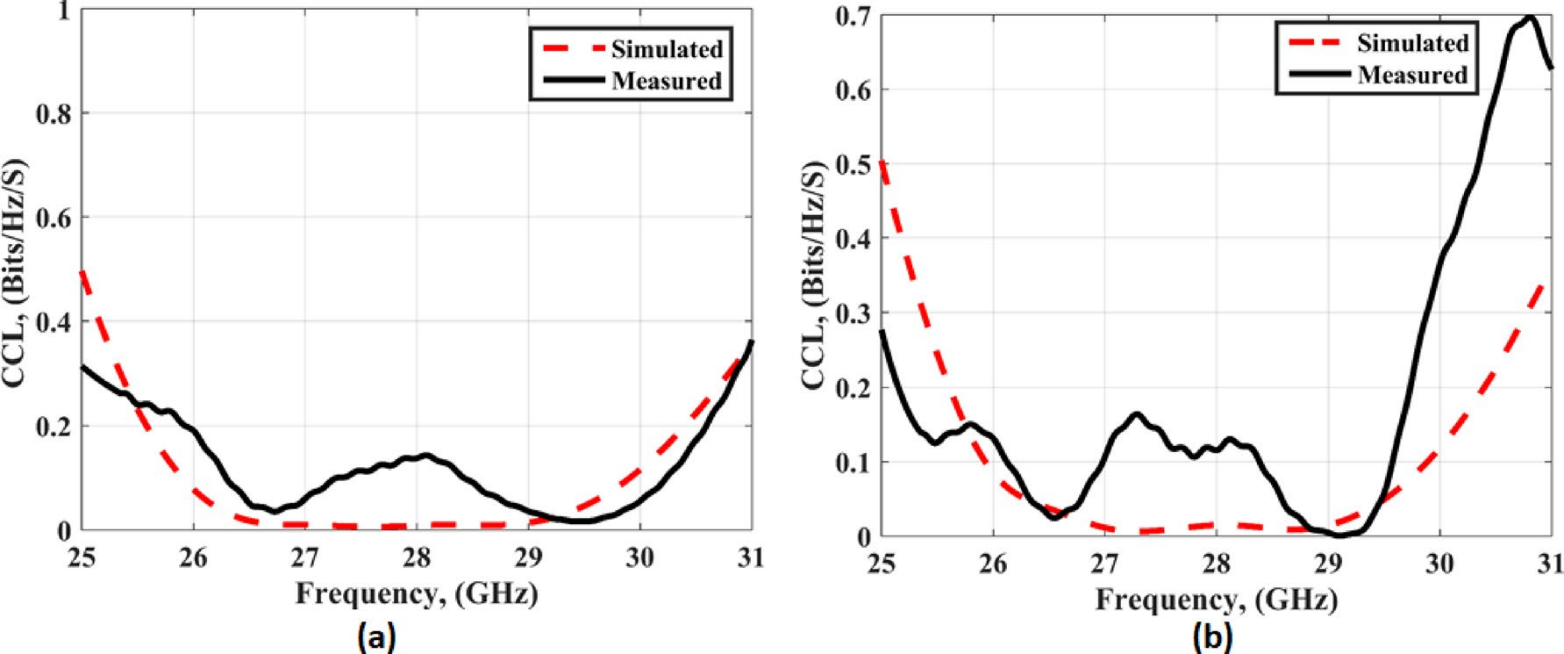
The CCL outcomes (**a**) without FSS (**b**) with FSS.

Figure 23 shows the value of the CCL is below 0.2bit/s/Hz in both cases with and without FSS through the entire bandwidth. From the previous results, in conclusion, the proposed antenna demonstrates excellent MIMO performance.

**Fig. 24:**
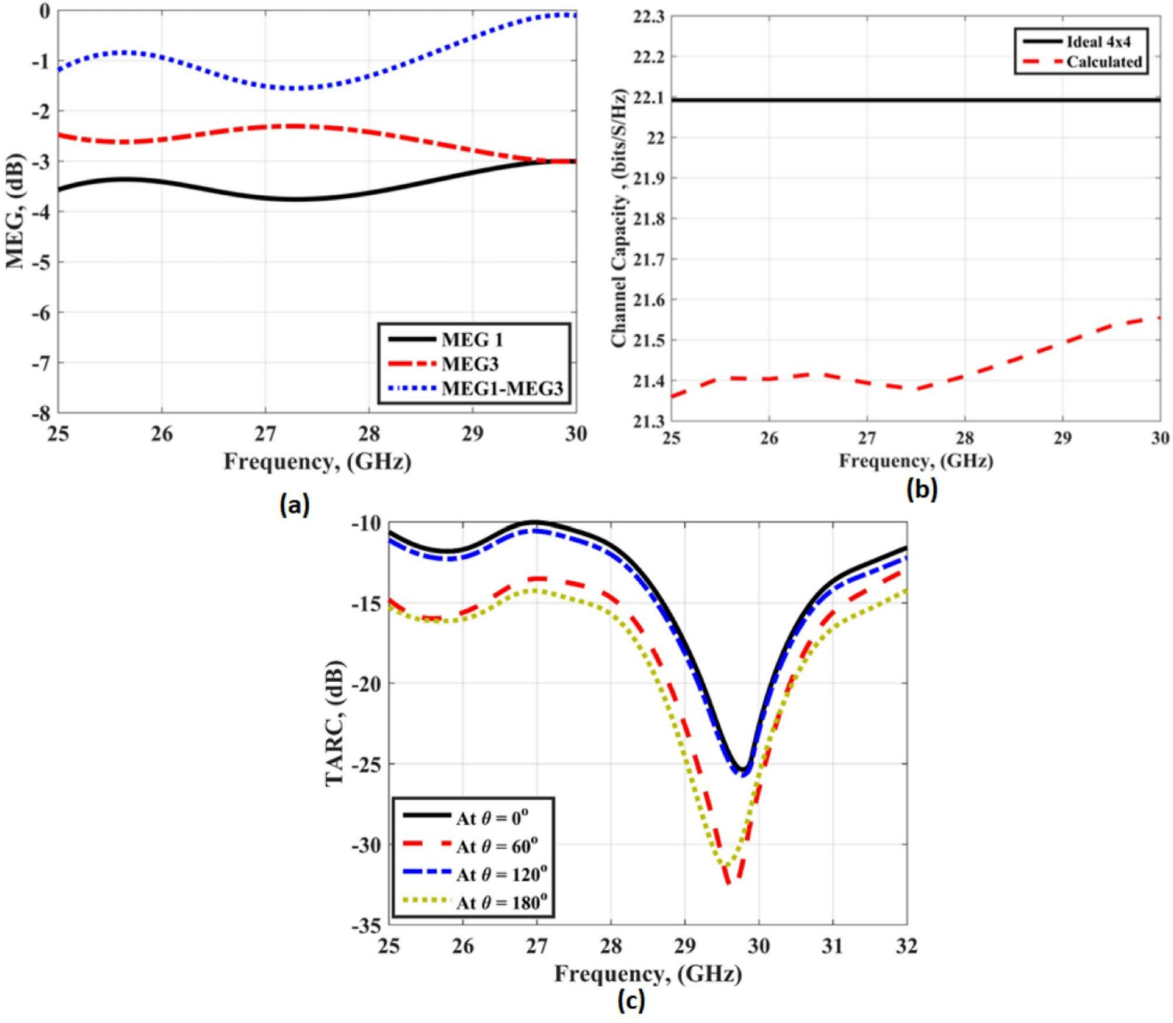
The antenna with FSS MIMO Parameters (**a**) MEG (**b**) Channel Capacity (**c**) TARC.

In^2,21,22^, the equations used to calculate the MEG, the channel capacity, and the TARC are utilized to indicate the MIMO performance as illustrated in Fig. 24. The MEG is calculated from Eq. (8) as^2^

8 $$MEG=0.5\left[ {1 - \sum\limits_{{j=1}}^{N} {{{\left| {{S_{ij}}} \right|}^2}} } \right].$$

The differences between the MEG at port 1 and port 3 equal a value lower than − 3dB within the desired frequency band as illustrated in Fig. 24(a).

The channel capacity for ideal and calculated quad ports is calculated from Eq. (9) as^22^

9 $$c=K{\log _2}\left[ {\det \left[ I \right]+\eta \frac{{SNR}}{K}\left[ H \right]\left[ {{H^*}} \right]} \right].$$

The I, SNR, K, H and H* are the identity matrix, signal to noise ratio, rank of the matrix, channel matrix and the Hermitian transpose matrix, respectively. The value of the channel capacity equal around 22.1 bits/s/Hz and 21.4 bits/s/Hz, for ideal and simulated outcomes, respectively as displayed in Fig. 24(b). Finally, the TARC is calculated from Eq. (10) as^22^

10 $$TARC=\frac{{\sqrt {\sum\limits_{{i=1}}^{N} {{{\left| {{S_{i1}}+\sum\limits_{{m=2}}^{N} {{S_{im}}{e^{j{\theta _{m - 1}}}}} } \right|}^2}} } }}{{\sqrt N }}.$$

The TARC achieved value lower than − 10 dB within the desired frequency band with around the same results at different phases as shown in Fig. 24(c).

This text summarizes a comparison of antenna designs based on Table 1 with designs introduced in^2,3,14–18^, and^24–28^ highlighting that the suggested MIMO antenna design shows good performance metrics. The statement emphasizes that the performance is suitable for 5G networks, making the design highly recommended for such applications.

**Table 1:** The comparison between the proposed MIMO structure with other designs.

Ref		Size mm^2^	ε_*r*_/h(mm)	Fo (GHz) /BW (GHz)	Max Gain (dBi)/ improving method	Isolation (dB)	ECC/ DG (dB)	CCL	Ports
^2^		32** × **20	2.2/0.508	28/4.9	5.6	≥ 30	0.001/9.99	< 0.4	2
^3^		12.4** × **12.4	2.2/0.508	28/4.5	6.27	≥ 22	0.05/9.99	0.2	4
^14^		24.1** × **7	2.92/1.027	26–28/6.3	7.1/array	-	-	-	1
^15^		32** × **32	2.2/0.787	28/1.5	10.6/array	≥ 30	< 0.001/ >9.99	-	4
^16^		31.2** × **31.2	2.2/1.57	25.4/5.68	6.4/ EBG	≥ 15	0.03/-	-	8
^17^		36.2** × **36.2	3.55/5	38/3	8.2/FSS	≥ 25	< 0.005/ >9.99	< 0.4	4
^18^		24** × **24	2.2/6.3	28/4.16	8.3–11.4/PSR	≥ 30	< 0.004/9.99	< 0.4	4
^24^		11 × 20.5	2.2/0.254	29/10	5.9	≥ 25	< 0.036/9.99	< 0.4	2
^25^		10 × 28	2.2/0.78	26.8/0.6–39.8/1.54	7.27–7.45	≥ 30	< 0.03/ 9.99	< 0.35	2
^26^		12 × 11.6	2.2/0.508	27.1/ 1.9 -48.7/2.8	6	≥ 27	< 10^− 6^ /9.99	< 0.3	4
^27^		15 × 15	2.2/0.8	39.5/1.7	5.98	20	< 0.001	0.37	4
^28^		17.76 × 17.76	2.2/1.52	28/0.65 -38/0.46	8.9	> 68	< 0.003/10	-	4
**This work**	**Wo FSS**	**25.7 × 25.7**	**3.38/0.203**	**28/6**	**6.05**	≥ 22	**< 0.002/9.99**	**< 0.2**	**4**
	**With FSS**	**37.8 × 37.8**	**3.38/5.2**	**28/5**	**8/ FSS**	**≥ 22**	**< 0.002/9.99**	**< 0.2**	**4**

## Conclusions

A four ports-slotted MIMO antenna structure with a cross-shaped cut and loaded with FSS for a higher band of 5G communication systems is introduced. The suggested antenna, operating at 28 GHz, features a single unit with a microstrip feed line on one side and a full ground plane on the other, with a rectangular slot antenna. The antenna proposed here is placed on an RO 4003 C substrate with size of 25.7 × 25.7 mm^2^ operating through a band from 25.5 up to 30 GHz. The suggested antenna’s features, including return loss, peak gain, and radiation characteristic, have been presented and tested. A cross-shaped cut is made to enhance the isolation between the elements of the proposed antenna without increasing the antenna size. The proposed structure is also fabricated and measured. The results indicate that the proposed structure is worked from 25.5 GHz up to 30 GHz with insertion loss ≤ -22 dB and peak gain of around 8dBi. As well, The ECC, the DG, and the CCL are measured and achieved ≤ 0.002, ≥ 9.99 dB, and ≤ 0.2 bit/s/Hz, respectively. Both the measured and simulated findings are matched and good agreements have been achieved.

## Data Availability

All data generated or analysed during this study are included in this published article.
